# Non-communicable comorbidities in pulmonary tuberculosis and healthcare utilization: a cross-sectional study of 2021 Indonesian national health insurance data

**DOI:** 10.1186/s13690-024-01352-y

**Published:** 2024-08-19

**Authors:** Danik Iga Prasiska, Durga Datta Chapagain, Kennedy Mensah Osei, Vasuki Rajaguru, Sun Joo Kang, Tae Hyun Kim, Sang Gyu Lee, Whiejong Han

**Affiliations:** 1 Global Health Security, Graduate School of Public Health, Yonsei University, Seoul, South Korea; 2 Healthcare Management, Graduate School of Public Health, Yonsei University, Seoul, South Korea; 3 Department of Global Health Security and Infectious Disease Control, Graduate School of Public Health, Yonsei University, Seoul, South Korea; 4 Department of Preventive Medicine, College of Medicine, Yonsei University, Seoul, South Korea

**Keywords:** Comorbidity, Health, Insurance, Non-communicable, Service, Tuberculosis

## Abstract

**Background:**

Limited research exists on the comorbidity of pulmonary tuberculosis with non-communicable diseases (NCDs) and its implications for healthcare utilization in Indonesia. The lack of investigation into NCD comorbidity among pulmonary tuberculosis patients could adversely affect both the healthcare system and the national health insurance scheme. Understanding the NCD comorbidity among pulmonary tuberculosis patients, associated factors, and healthcare utilization is crucial for ensuring the effective and efficient delivery of health services.

**Method:**

This study utilized an observational cross-sectional design based on anonymized sample data from tuberculosis cases covered by Indonesia's National Health Insurance in 2021. Chi-square tests were employed to analyze dependent and independent variables, while unadjusted and adjusted logistic regressions were used to explore further associations.

**Results:**

The prevalence of NCD comorbidity in tuberculosis patients was 11.81%. Aged over 60 (aOR 5.16; [CI] 4.23—6.3), married (aOR 1.19; [CI] 1.05—1.34), and unemployed (aOR 1.27; [CI] 1.08—1.49) were associated with the NCD comorbidity in pulmonary tuberculosis patients. Factors associated with increased inpatient service utilization among pulmonary tuberculosis patients included aged over 60 (aOR 5.69; [CI] 4.81—6.74), male (aOR 1.32; [CI] 1.23—1.40), self-employment (aOR 1.42; [CI] 1.29—1.56), having insurance subsidized by central government (aOR 1.89; [CI] 1.73—2.08) or local government funds (aOR 1.75; [CI] 1.58—1.93), and having comorbidity non-communicable diseases (aOR 1.80; [CI] 1.66—1.96).

**Conclusion:**

Pulmonary tuberculosis patients exhibit a significant prevalence of NCD comorbidity, which substantially impacts healthcare utilization. Early detection and management of these conditions are critical to mitigate burdens on both the healthcare system and the financial sustainability of the national health insurance scheme. Integrating health services for tuberculosis and NCDs through bidirectional screening is essential for comprehensive patient care.

**Supplementary Information:**

The online version contains supplementary material available at 10.1186/s13690-024-01352-y.

**Table:** 

Text box 1. Contributions to the literature
• Limited evidence was available on the non-communicable disease comorbidity and its impact on health service utilization among pulmonary tuberculosis patients.
• The growing trend of non-communicable disease comorbidity among pulmonary tuberculosis patients could threaten the stability and financial measures of Indonesia's National Health Insurance scheme.
• Effective and efficient integration of health services, including bidirectional screening for for both tuberculosis and non-communicable diseases, is essential.

## Background

 Globally, Tuberculosis (TB) is the second leading deadliest infectious disease. It was estimated that a quarter of the global population is estimated to have been infected with TB bacteria, and 5–10% of people infected with TB will eventually get symptoms and develop TB disease [1]. WHO reported that a total of 1.3 million people died from TB in 2022 (including 167,000 people with HIV) [1]. Based on WHO estimation, Indonesia is ranked as the second country with the highest TB burden. In 2021, the TB incidence rate is 354 per 100.000 populations, the TB incidence rate among HIV^+^ is 8 per 100.000 populations, and the TB mortality rate is 52 per 100.000 populations [2]. Several studies reported growing evidence of the association between tuberculosis and Non-Communicable Diseases (NCD) [3, 4], contributing to the double burden disease in the most of Low-Middle Income Countries (LMICs) including Indonesia.

In 2019, WHO reported that just over 15% of people with TB were estimated to have diabetes globally [5]. In addition to diabetes, several studies reported that people with tuberculosis are prone to developing NCD comorbidity. The association between chronic kidney disease (CKD) and TB is well-documented, several studies reported that people who had CKD were more susceptible to tuberculosis [6, 7]. Chronic Obstructive Pulmonary Disease (COPD) was also reported as one of the significant comorbidities of tuberculosis, following diabetes [8]. A systematic review study indicated that patients with TB have an increased risk of cardiovascular diseases (CVDs) [9]. Other systematic and meta-analysis studies reported that individuals with TB have an increased risk of both pulmonary and non-pulmonary cancers [10]. It was also reported that people with TB showed evidence of the effects of mental health on TB outcomes, depression, anxiety poor social support, and stigma affect the mental well-being of individuals with TB across the globe [11]. Overall, it was well noted that globally, individuals with TB are predisposed to developing NCD as a comorbidity.

A recently developed age-structured TB-Diabetes Mellitus (DM) dynamic mathematical model reported that, one in five TB disease cases and one in four TB-related deaths are attributed to DM in Indonesia, and by 2050, they will increase to one in four and one in three, respectively [12]. National-level policy for screening for DM in TB exists in Indonesia. Yet, there has been no effort to actively screen for TB in people with DM on care [13]. While the burden of DM among Pulmonary TB patients has been investigated, the burden of other NCD comorbidity in patients with tuberculosis remains under-investigated. There is a limited study of TB with NCD comorbidity and its impact on healthcare utilization in Indonesia due to the lack of integration into the surveillance system [14]. Several key stakeholders in Indonesia stated that most current health services and programs for adolescents do not routinely address NCD risks [15]. The absence of investigation of NCD comorbidity amongst patients with TB may impact negatively the health system and hinder the achievement of Sustainable Development Goals (SDGs) [16].

This study linked concurrent health issues arising from the combined impact of certain NCD and TB, necessitating a shift towards public health collaboration and preventive strategies. Understanding the patterns of comorbidity involving pulmonary TB is crucial for ensuring effective and efficient health service delivery. Therefore, the primary objective of this research was to identify comorbidities including Diabetes Mellitus (DM), Chronic Obstructive Pulmonary Disease (COPD), cardiovascular diseases (CVD), Cancer, Chronic Kidney Disease (CKD), and Mental Health disorders, in pulmonary TB patients among Indonesia's National Health Insurance (NHI) users. The secondary objectives were to determine factors associated with NCD comorbidities and to assess healthcare utilization in these patients.

## Method

This study was reported using the Strengthening the Reporting of Observational Studies in Epidemiology (STROBE) guidelines (Supplementary Material). An observational cross-sectional study design was employed, based on the anonymous sample dataset of contextual TB among Indonesia National Health Insurance users in 2021. We utilized the referral care dataset (primary and secondary diagnoses) linked to the membership dataset. An anonymous Indonesia National Health Insurance sample data is available for the public upon request at https://data.bpjs-kesehatan.go.id/ , provided by the Social Security Administration for Health or *Badan Penyelenggaran Jaminan Sosial (BPJS) Kesehatan*. Data analysis included six major NCDs using the International Classification of Disease (ICD)-10 codes (Supplementary Material). Missing Not at Random (MNAR) was used to handle missing data. To ensure an impartial estimation, non-informative data were deleted. The Receiver Operating Characteristic (ROC) curve and Area Under Curve (AUC) were used to evaluate the fit of the analyzed model (Supplementary Material).

### Dependent variables

This study included all the participants diagnosed with Pulmonary TB as the primary diagnosis. The TB patients who had one or more diagnoses of NCD were classified as Pulmonary-TB patients with NCD or comorbidity. Healthcare utilization was determined by inpatient and outpatient services used by the pulmonary TB patients with or without NCD comorbidity, in 2021.

### Independent variables

The sociodemographic characteristics were determined by age group, sex, marital status, residential location, and insurance segmentation. Marital status was categorized into single, married, and divorced. Residential location was categorized based on the province in six major islands regions, namely Sumatera, Java, Bali, NTT, & NTB, Kalimantan, Sulawesi, and Maluku & Papua. Insurance segmentation was categorized into five groups including, unemployment (the individuals who currently not engaged in any employment), Subsidized Central (subsidized by central government fund), Subsidized Local (subsidized by local /regional government fund), Self Employed (individuals who pay the premium fully by themselves), and Employee (individuals whose premium is paid by the employers with contributions from employees).

### Data analysis

The data were analyzed in three steps. First, all of the data were analyzed with descriptive statistics to produce tabulation frequency and percentage for each variable. Second, the differences in patient characteristics were examined using a chi-square analysis. Lastly, logistic regression with the backward method was employed to examine the relationship between the TB patients with comorbidities and explanatory variables, while controlling for potential confounders. Adjusted odds ratios (aORs) with 95% confidence intervals (CIs) were reported. All the data analysis was conducted using SPSS version 27.0 and *p*-value < 0.005 was considered statistically significant.

### Socio-demographic characteristics

Table 1 summarizes the sociodemographic characteristics and associations of the study population. A total of 27,449 pulmonary tuberculosis patients were included in the study. The majority of them were aged 40–60 years (30.8%), male (57.5%), and married (58.99%). Most of patients resided in Sumatera Island (33.9%) and were employed (27.7%). All characteristics showed statistically significant levels at *p* < 0.001.

**Table 1:** Sociodemographic characteristics and association of pulmonary tuberculosis with non-communicable disease comorbidity among Indonesian National Health Insurance Users, 2021

Variable		Total		NCD comorbidity status				*p-*value
				No		Yes		
		N	%	N	%	N	%	
Age	< 18	4,683	(17.0)	4,497	(18.5)	186	(5.7)	< 0.001^*^
	18 to 40	7,765	(28.2)	7,293	(30.0)	472	(14.5)	
	< 40 to 60	8,481	(30.8)	7,229	(29.8)	1,252	(38.5)	
	> 60	6,570	(23.8)	5,231	(21.5)	1,339	(41.2)	
Sex	Male	15,803	(57.4)	13,847	(57.1)	1,956	(60.2)	< 0.001^*^
	Female	11,696	(42.5)	10,403	(42.9)	1,293	(39.8)	
Marital status	Single	9,853	(35.8)	9,274	(38.2)	579	(17.8)	< 0.001^*^
	Married	16,222	(58.9)	13,813	(56.9)	2,409	(74.1)	
	Divorced	1,424	(5.2)	1,163	(4.8)	261	(8.0)	
Residential location	Sumatera	9,330	(33.9)	8,144	(33.6)	1,186	(36.5)	< 0.001^*^
	Java	7,644	(27.8)	6,787	(27.9)	857	(26.4)	
	Bali, NTT, & NTB	1,881	(6.8)	1,702	(7.0)	179	(5.5)	
	Kalimantan	3,244	(11.8)	2,861	(11.8)	383	(11.8)	
	Sulawesi	3,334	(12.1)	2,914	(12.0)	420	(12.9)	
	Maluku & Papua	2,064	(7.5)	1,840	(7.6)	224	(6.9)	
Segmentation	Unemployed	1,348	(4.9)	1,053	(4.34)	295	(9.1)	< 0.001^*^
	Subsidized Central	6,906	(25.1)	6,082	(25.1)	824	(25.4)	
	Subsidized Local	5,039	(18.3)	4,405	(18.2)	634	(19.5)	
	Self Employed	6,581	(23.9)	5,789	(23.8)	792	(24.4)	
	Employee	7,625	(27.7)	6,921	(28.5)	704	(21.7)	
Healthcare utilization	Inpatient	5,491	(20.0)	4,410	(18.3)	1,081	(31.7)	< 0.001^*^
	Outpatient	22,008	(80.0)	19,677	(81.7)	2,331	(68.3)	

^*^Indicates a significance level less than 0.05

**Fig. 1:**
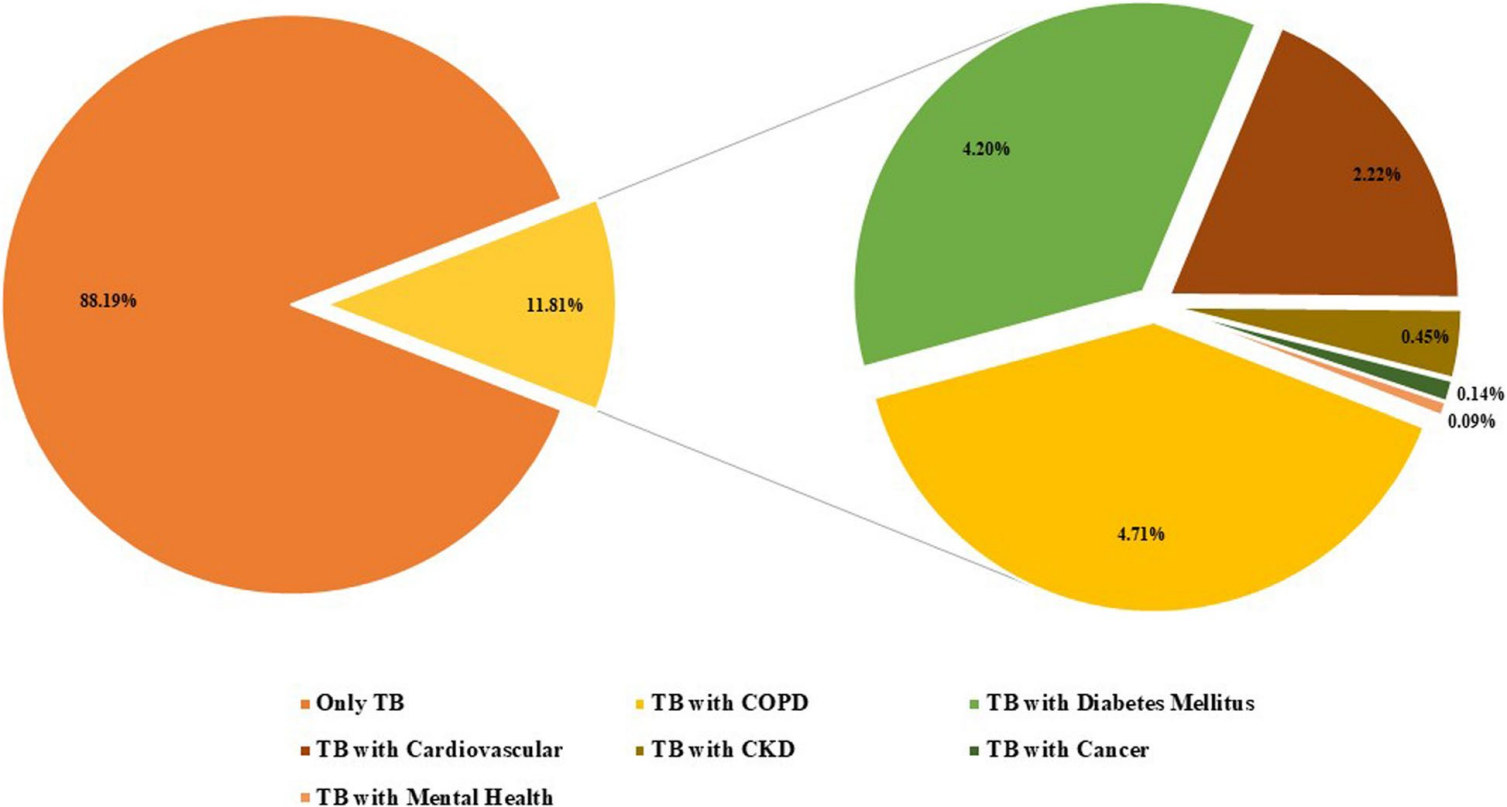
Distribution of pulmonary tuberculosis patients with non-communicable disease comorbidity among Indonesian National Health Insurance Users, 2021

There were 3,249 (11.81%) patients primarily diagnosed with pulmonary TB has one of five major NCD comorbidities. Chronic Obstructive Pulmonary Disease (COPD) accounted for the highest NCD comorbidity (4.71%), followed by Diabetes Mellitus (4.2%) and Cardiovascular Disease (2.22%). Mental Health disorders (0.09%) were identified as the least NCD comorbidity among pulmonary tuberculosis patients (Fig. 1). The prevalence of NCD comorbidity predominantly occurs in the elderly patients (41.2%), male patients (60.2%), those who were married (74.1%), residents of Sumatera (36.5%), and patients supported by subsidies from the central government fund (25.4%).

Table 2 shows that among Indonesian National Health Insurance users who entered referral care in 2021, identified that 22,008 (80.0%) pulmonary tuberculosis patients utilized outpatient services, while 5,491 (20.0%) utilized inpatient services. It was observed that the majority of users of both outpatient (29.7%) and inpatient (35.4%) services were people in their forty to sixty years old. A similar  pattern was observed for the sex, marital status, residential location, and comorbidity status: males, married, residents of Sumatra, and patients with only diagnosed with pulmonary tuberculosis were predominantly using both outpatient and inpatient services. In terms of segmentation, it was noticed that employees (30.2%) were most likely to utilize outpatient service, while inpatient service was most commonly utilized by patients subsidized by central government funds (31.96%).

**Table 2:** Sociodemographic characteristics and association with health service Utilization among Indonesian National Health Insurance Users with Pulmonary Tuberculosis, 2021

Variable		Healthcare utilization						*p-*value
		n (%)		Outpatient		Inpatient		
				n (%)		n (%)		
Age	< 18	4,683	(17.0)	4,452	(20.2)	231	(4.2)	< 0.001^*^
	18 to 40	7,765	(28.2)	6,225	(28.3)	1,540	(28.0)	
	< 40 to 60	8,481	(30.8)	6,535	(29.7)	1,946	(35.4)	
	> 60	6,570	(23.9)	4,796	(21.8)	1,774	(32.3)	
Sex	Male	15,803	(57.5)	12,328	(56.0)	3,475	(63.3)	< 0.001^*^
	Female	11,696	(42.5)	9,680	(43.9)	2,016	(36.7)	
Marital Status	Single	9,853	(35.8)	8,467	(38.5)	1,386	(25.2)	< 0.001^*^
	Married	16,222	(58.9)	12,485	(56.7)	3,737	(68.0)	
	Divorced	1,424	(5.2)	1,056	(4.8)	368	(6.7)	
Residential Location	Sumatera	9,330	(33.9)	7,359	(33.4)	1,971	(35.9)	< 0.001^*^
	Java	7,644	(27.8)	6,515	(29.6)	1,129	(20.5)	
	Bali, NTT, & NTB	1,881	(6.8)	1,480	(6.7)	401	(7.3)	
	Kalimantan	3,244	(11.8)	2,621	(11.9)	623	(11.3)	
	Sulawesi	3,334	(12.1)	2,414	(10.9)	920	(16.7)	
	Maluku & Papua	2,064	(7.5)	1,617	(7.3)	447	(8.1)	
Segmentation	Unemployed	1,348	(4.9)	1,068	(4.8)	280	(5.10)	< 0.001^*^
	Subsidized Central	6,906	(25.1)	5,151	(23.4)	1,755	(31.9)	
	Subsidized Local	5,039	(18.3)	3,847	(17.5)	1,192	(21.7)	
	Self Employed	6,581	(23.9)	5,290	(24.0)	1,291	(23.5)	
	Employee	7,625	(27.7)	6,652	(30.2)	973	(17.7)	
Comorbidity Status	No	24,250	(88.2)	19,811	(90.0)	4,439	(80.8)	< 0.001^*^
	Yes	3,249	(11.8)	2,197	(9.9)	1,052	(19.2)	

^*^Indicates a significance level less than 0.05

### Factor associated with non-communicable disease comorbidity in pulmonary tuberculosis patient

Patients over 18 years old (18 to 40 (aOR 1.47; 1.22—1.76), 40 to 60 (aOR 3.62; 2.98—4.39), and > 60 (aOR 5.16; 4.23—6.3)), married (aOR 1.19; 1.05—1.34), and unemployed (aOR 1.27; 1.08—1.49) were associated with the NCD comorbidity in pulmonary tuberculosis patient. Residing in Bali, NTT, & NTB (aOR 0.72; 0.58—0.88) was inversely associated with the NCD comorbidity in pulmonary tuberculosis patients.

### Factors associated with health service utilization

TB Patients aged > 60 (aOR 5.69; 4.81—6.74), males (aOR 1.32; 1.23—1.40), self-employed (aOR 1.42; 1.29—1.56), insurance subsidized by central (aOR 1.89; 1.73—2.08) and local government fund (OR 1.75; 1.58—1.93), and having NCD comorbidities (aOR 1.80; 1.66—1.96) were associated with the inpatient service utilization in pulmonary tuberculosis patients. Residing in Java (aOR 0.64; 0.56—0.73) was inversely associated with inpatient service utilization in pulmonary tuberculosis patients and was statistically significant (*p* < 0.001) (Table 3).

**Table 3:** Logistic regression of pulmonary tuberculosis with non-communicable disease comorbidity and health service utilization among Indonesian National Health Insurance Users, 2021

	Variable	TB with NCD comorbidity				Health service utilization			
		aOR	CI 95%		***p-***value	aOR	CI 95%		***p-***value
			Lower	Upper			Lower	Upper	
Age	< 18	1.00				1.00			
	18 to 40	1.46	1.22	1.76	< 0.001^*^	4.53	3.90	5.26	< 0.001^*^
	< 40 to 60	3.62	2.97	4.39	< 0.001^*^	4.96	4.21	5.84	< 0.001^*^
	> 60	5.16	4.23	6.31	< 0.001^*^	5.69	4.81	6.75	< 0.001^*^
Sex	Male	0.99	0.92	1.08	0.96	1.32	1.24	1.40	< 0.001^*^
	Female	1.00				1.00			
Marital status	Single	1.00				1.00			
	Married	1.18	1.04	1.34	0.008^*^	0.97	0.89	1.07	0.615
	Divorced	1.20	0.99	1.45	0.052	1.09	0.94	1.27	0.256
Residential location	Sumatera	1.07	0.91	1.25	0.396	0.90	0.80	1.02	0.101
	Java	0.98	0.83	1.15	0.847	0.64	0.56	0.72	< 0.001^*^
	Bali NTT NTB	0.71	0.58	0.88	0.002^*^	0.86	0.74	1.01	0.069
	Kalimantan	1.04	0.87	1.25	0.650	0.89	0.77	1.03	0.126
	Sulawesi	0.97	0.82	1.16	0.789	1.22	1.06	1.39	0.004^*^
	Maluku Papua	1.00				1.00			
Segmentation	Unemployed	1.27	1.07	1.49	0.005^*^	1.11	0.95	1.30	0.181
	Subsidized Central	1.02	0.91	1.14	0.715	1.89	1.73	2.08	< 0.001^*^
	Subsidized Local	1.10	0.98	1.25	0.086	1.75	1.58	1.93	< 0.001^*^
	Self Employed	0.98	0.87	1.09	0.726	1.42	1.29	1.56	< 0.001^*^
	Employee	1.00				1.00			
Comorbidity	No	-	-	-	-	1.00			
	Yes	-	-	-	-	1.80	1.65	1.96	< 0.001^*^

^*^Indicates a significance level less than 0.05

### Non-communicable disease comorbidity and inpatient health service utilization

Tuberculosis patients with NCD comorbidities have the likelihood to access more inpatient care compared to those without NCD comorbidities, except for individuals diagnosed  with Chronic Obstructive Pulmonary Disease (aOR 0.25; 0.20—0.31), Cancer (aOR 1.62; 0.82-3.18), and Mental Health disorders (aOR 2.47; 0.97—6.32) (Table 4).

**Table 4:** Logistic regression of non-communicable disease comorbidity with inpatient health service utilization among Indonesian National Health Insurance Users with Pulmonary Tuberculosis, 2021

NCD comorbidity	Status	OR (95% CI)	*p-*value	aOR (95% CI)	*p-*value
Diabetes mellitus	No	1.00	< 0.001^*^	1.00	< 0.001^*^
	Yes	5.42 (4.81—6.12)		4.98 (4.40—5.64)	
Chronic obstructive pulmonary disease	No	1.00	< 0.001^*^	1.00	< 0.001^*^
	Yes	0.32 (0.26—0.39)		0.25 (0.20—0.31)	
Cardiovascular-diseases	No	1.00	< 0.001^*^	1.00	< 0.001^*^
	Yes	2.63 (2.23—3.10)		2.07 (1.75—2.45)	
Cancer	No	1.00	0.032^*^	1.00	0.161
	Yes	2.08 (1.07—4.08)		1.62 (0.82—3.18)	
Chronic kidney disease	No	1.00	< 0.001^*^	1.00	< 0.001^*^
	Yes	3.50 (2.46—4.98)		3.03 (2.11—4.34)	
Mental health disorders	No	1.00	0.264	1.00	0.058
	Yes	1.65 (0.68—3.98)		2.47 (0.97—6.32)	

*OR* Univariate Odds Ratio, *aOR* Adjusted for sex, age, and marriage status

^*^Indicates a significance level less than 0.05

## Discussion

This study aimed to investigate the factors associated with NCD comorbidities and the health service utilization in Pulmonary Tuberculosis patients enrolled in Indonesia's National Health Insurance. A total of 27,449 Pulmonary Tuberculosis patients were identified, with 11.8% of them having NCD. Our study found that age is one of the predictors of NCD comorbidity in Pulmonary Tuberculosis patients, it was clear that older patients were more likely to have NCD comorbidity compared with a younger patient, this result is in line with finding from a study in Gabon [4]. In that study, individuals aged over 55 years was associated with diabetes (aOR 6·99; 2·10–25·44) and hypertension (aOR 7.48; 2·36–25·30) in multivariable analysis. Similiarly, a study in Southern Ethiopia reported the same result that individuals aged over 50 are at 22.13 (3.46, 141.41) times higher risk than those aged 20–34 years of developing TB with multi-comorbid NCD [17]. Addresing health in the elderly presents a public health challenge, the elderly are at greater risk of vulnerability due to their nature of physical and functional health risks [18].

Contrary to the previous study [19], this study did not find an association between males and tuberculosis NCD comorbidity. Our study reported that married patients are more likely to have NCD comorbidity, which is consistent with findings from a study conducted in South Africa. However, there is a different result in divorced patients [19]. Previous studies reported that the highest prevalence of Tuberculosis was observed in Sumatera [20], and our study also reported the same finding. It was also noticed that the highest prevalence of NCD comorbidity in pulmonary tuberculosis patients was also in Sumatra followed by Java (Table 1). This could have been a result of the extensive availability of healthcare facilities in Sumatra and Java islands in comparison to other islands [20].

The highest proportion of NCD comorbidity among TB patients was reported in poor and underprivileged individuals with the support of the central government. Previous studies support the findings that populations with lower socioeconomic status are at an elevated risk of developing NCD—diabetes, stroke, myocardial infarction (heart attack), and cancer [21, 22]. An unhealthy lifestyle especially food choices may have a high contribution to the NCD. Industrialization and global food market dynamics impact food choices amidst constraints such as limited household income, time availability, and household and community resources. Additionally, risk factors for non-communicable diseases (NCDs) are exacerbated by low household income and the poverty of environments inhabited by low-income individuals, limiting opportunities for physical activity among those in sedentary occupations. These circumstances underscore the minimal control individuals often have over their diet and exercise routines [23].

Our study showed slightly higher COPD prevalence (4.71%) among active pulmonary compared to the study conducted in Tanzania. In the Tanzanian study it was reported that among COPD patients there was a 10% prevalence of patients with a history of pulmonary tuberculosis but currently inactive or already cured and 3% prevalence of patient with current active tuberculosis suspected to have COPD [24]. A meta-analysis study reported that individuals with prior infection of Pulmonary tuberculosis have a greater risk of developing Chronic Obstructive Pulmonary Disease [25]. Based on WHO estimation, Indonesia is ranked second with the highest TB burden. In 2021, the TB incidence rate is 354 per 100.000 population and the TB mortality rate is 52 per 100.000 population [2]. Based on the WHO estimation, it is known that Indonesia has a greater tuberculosis burden compared to the country in the African region. It was noted that post-tuberculosis lung damage is a recognized consequence of pulmonary TB [26] and is positively associated with pulmonary function impairment, leading to frequent respiratory symptoms [27]. However, the interrelationship between TB and COPD is very complex [28]. Our study reported that the prevalence of Diabetes Mellitus comorbidity in pulmonary tuberculosis patients was far less than in a previous study done in Indonesia [12, 29]. The difference in the data collection method and study population could explain the variations in results across the studies.

Our study only accounted for the pulmonary tuberculosis patients who accessed referral care using the national health insurance scheme in 2021. It should be noted that the Indonesia government has already enhanced the screening and treatment of pulmonary tuberculosis together aligned with Diabetes Mellitus screening in the primary health center [2], even though these efforts are still not evenly distributed throughout Indonesia.

Cardiovascular disease was well known as the secondary disease that could be developed in people with active pulmonary tuberculosis. Approximately 60% of patients with TB have a cardiovascular disease [30]. Yet, commonly underestimated due to a lack of suspicion [31]. The association between tuberculosis and lung cancer has been discovered, it is stated that the mycobacterium tuberculosis infection accelerates the development of lung cancer [32]. Studies in South Korea reported that younger patients with TB have a more substantial likelihood of lung cancer than older patients with pulmonary TB [33]. A Cohort study done in the United Kingdom and China reported that chronic kidney disease (CKD) is associated with an increased risk of active Tuberculosis [34, 35]. Mental health was reported as the comorbidity of Tuberculosis. According to the WHO, Tuberculosis patients have a higher risk of mental health conditions, which can negatively impact tuberculosis treatment, quality of life, and other health and social outcomes [36].

It is noted that the patients with NCD comorbidity require more inpatient health services compared to the people without NCD comorbidity. A previous study reported that the existence of NCD comorbidity in Pulmonary Tuberculosis patients is highly associated with decreased physical health [19]. A systematic review has shown  that individuals with multimorbidity conditions require more outpatient and inpatient care [37], which is also supported by our study. Additionally, It was observed that the subsidized patients significantly contribute to the utilization of inpatient health services. This poses a threat to the National Health Insurance scheme, as it has been reported to consistently incur severe deficits [38].

Tuberculosis and non-communicable diseases share a similar multiple risk factor [39], suggesting that integrating both Tuberculosis and NCD screening and treatment programs could potentially enhance the treatment outcomes of both tuberculosis and NCD simultaneously. Indonesia has developed a national screening program that integrates tuberculosis with Diabetes Mellitus [2, 13]. The effective result of the integration of tuberculosis and diabetes mellitus was reported in a study conducted in Jakarta, Indonesia, and Luanda, Angola, where receiving Diabetes Mellitus treatment was associated with a higher likelihood of completing TB treatment [40, 41]. However, a systematic review analysis concluded that the integration of tuberculosis and Diabetes Mellitus was weak to enhance both treatment outcomes [40]. Another qualitative study in Yogyakarta, Indonesia reported that there were barriers existing in delivering the tuberculosis–diabetes mellitus integration program. The barriers were health services-related barriers, patient-related barriers, and health provider-patient interaction-related barriers. To enhance the health outcomes of the program, effort to minimize the existing barriers should be considered [42].

The integration impact of tuberculosis–diabetes mellitus has been proven to some extent to enhance the program outcomes. However, the integration program on other NCD comorbidities was nowhere to be found. Given the numbers needed to test to detect a new case for each of the non-communicable diseases, it seems feasible to incorporate routine screening of pulmonary tuberculosis and secondary prevention of common NCD. However, it should be noted that systematic screening for non-communicable diseases during pulmonary tuberculosis care would require capacity building and a more inclusive focus on the patient’s health outcomes. The national tuberculosis control program in many LMICs integrated successfully the screening of HIV in tuberculosis patients. Therefore, extending the program to NCD comorbidity may not be a huge challenge to provide a new scheme in health service delivery.

Our findings highlight the crucial significance of enhancing relationships between TB and NCD control programs to strengthen the management of TB-NCD comorbidities. Integrated care can significantly improve the efficiency of health service delivery, particularly in resource-constrained settings. There is an urgent need to realign and improve healthcare system responses to establish effective screening programs that prevent and control NCDs using cost-efficient interventions and well-structured, integrated methods to provide high-quality primary care. Equal preventative and treatment approaches are also essential. The expanding prevalence of double-burden diseases must be recognized as a potential hazard to the stability of Indonesia's national health insurance scheme.

The strength of our study was the enrolment of a large number of participants from the NHI sample data that could cover the national sampling coverage. The diagnosis to confirm the patient was based on the laboratory and medical confirmed ICD X code, therefore misclassification could be minimalized. There are some limitations of our study due to the nature of using secondary data, some other related variables correlation could not be addressed such as the severity level of the diseases and health behavior of the patients. Indonesia's health insurance system uses a referral policy, in which patients should receive treatment from the primary level before going to the referral facility [43]. Therefore, most tuberculosis patients who do not have any severe complication condition are less likely to receive treatment in a referral hospital. With this fact, our study may only report a small part of the tuberculosis patients with non-communicable disease comorbidity. Consequently, the findings may not represent the entire population of pulmonary tuberculosis patients in Indonesia.

## Conclusion

Pulmonary tuberculosis patients have a considerable NCD comorbidity and have a significant impact on health service utilization. The growing trend of double burden disease should be acknowledged as it could threaten the stability of the national health insurance scheme. Early detection and management care should be considered critical to prevent overburden not only in the health system but also in the financial measure of the national health insurance scheme. Integration of health services for both tuberculosis and non-communicable diseases through bidirectional screening is essential.

### Supplementary Information

Supplementary Material 1

## Data Availability

Social Security Administration for Health or Badan Penyelenggaran Jaminan Sosial (BPJS) Kesehatan provided an anonymous Indonesia's National Health Insurance sample data. This data is available for the public upon request at https://data.bpjs-kesehatan.go.id/.

## Abbreviations

AUC: Area Under Curve
COPD: Chronic Obstructive Pulmonary Diseases
CVD: Cardiovascular Diseases
DM: Diabetes Mellitus
HIV: Human Immuno-deficiency Virus
ICD: International Classification of Diseases
LMICs: Low-Middle Income Countries
NCD: Non-Communicable Diseases
NHI: National Health Insurance
ROC: Receiver Operating Characteristic
TB: Tuberculosis
