# Supplementary material for: Non-communicable comorbidities in pulmonary tuberculosis and healthcare utilization: a cross-sectional study of 2021 Indonesian national health insurance data
Source: Arch Public Health. 2024 Aug 19;82:127. doi: 10.1186/s13690-024-01352-y (PMC11331679; doi:10.1186/s13690-024-01352-y)
Supplement: Supplementary file 1 — Supplementary Material 1 [file 13690_2024_1352_MOESM1_ESM.docx]

**Supplementary File**

Strengthening The Reporting of Observational Studies in Epidemiology Checklist of Non-communicable Comorbidities in Pulmonary Tuberculosis and Healthcare Utilization: A Cross-Sectional Study of 2021 Indonesian National Health Insurance Data

|  | Item No | Recommendation | Page No |
| --- | --- | --- | --- |
| **Title and abstract** | 1 | (*a*) Indicate the study’s design with a commonly used term in the title or the abstract | 1-2 |
|  |  | (*b*) Provide in the abstract an informative and balanced summary of what was done and what was found | 2 |
| Introduction | | |  |
| Background/rationale | 2 | Explain the scientific background and rationale for the investigation being reported | 3 |
| Objectives | 3 | State-specific objectives, including any prespecified hypotheses | 4 |
| Methods | | |  |
| Study design | 4 | Present key elements of study design early in the paper | 4 |
| Setting | 5 | Describe the setting, locations, and relevant dates, including periods of recruitment, exposure, follow-up, and data collection | 4 |
| Participants | 6 | (*a*) Give the eligibility criteria and the sources and methods of selection of participants | 4 |
| Variables | 7 | Clearly define all outcomes, exposures, predictors, potential confounders, and effect modifiers. Give diagnostic criteria, if applicable | 4-5 |
| Data sources/ measurement | 8 | For each variable of interest, give sources of data and details of methods of assessment (measurement). Describe comparability of assessment methods if there is more than one group | *4-5* |
| Bias | 9 | Describe any efforts to address potential sources of bias | 4 |
| Study size | 10 | Explain how the study size was arrived at | 5 |
| Quantitative variables | 11 | Explain how quantitative variables were handled in the analyses. If applicable, describe which groupings were chosen and why | 5 |
| Statistical methods | 12 | (*a*) Describe all statistical methods, including those used to control for confounding | 5 |
|  |  | (*b*) Describe any methods used to examine subgroups and interactions | NA |
|  |  | (*c*) Explain how missing data were addressed | 4 |
|  |  | (*d*) If applicable, describe analytical methods taking account of sampling strategy | NA |
|  |  | (*e*) Describe any sensitivity analyses | NA |
| Results | | |  |
| Participants | 13 | (a) Report numbers of individuals at each stage of study—eg numbers potentially eligible, examined for eligibility, confirmed eligible, included in the study, completing follow-up, and analyzed | 5 |
|  |  | (b) Give reasons for non-participation at each stage | NA |
|  |  | (c) Consider the use of a flow diagram | NA |
| Descriptive data | 14 | (a) Give characteristics of study participants (eg demographic, clinical, social) and information on exposures and potential confounders | 4 |
|  |  | (b) Indicate the number of participants with missing data for each variable of interest | NA |
| Outcome data | 15 | Report numbers of outcome events or summary measures | 5-9 |
| Main results | 16 | (*a*) Give unadjusted estimates and, if applicable, confounder-adjusted estimates and their precision (eg, 95% confidence interval). Make clear which confounders were adjusted for and why they were included | 8-9 |
|  |  | (*b*) Report category boundaries when continuous variables were categorized | 5-9 |
|  |  | (*c*) If relevant, consider translating estimates of relative risk into absolute risk for a meaningful period | NA |
| Other analyses | 17 | Report other analyses are done—eg analyses of subgroups and interactions, and sensitivity analyses. | NA |
| Discussion | | |  |
| Key results | 18 | Summarise key results concerning study objectives | 9,11 |
| Limitations | 19 | Discuss the limitations of the study, taking into account sources of potential bias or imprecision. Discuss both the direction and magnitude of any potential bias | 12 |
| Interpretation | 20 | Give a cautious overall interpretation of results considering objectives, limitations, multiplicity of analyses, results from similar studies, and other relevant evidence | 12 |
| Generalisability | 21 | Discuss the generalisability (external validity) of the study results | 12 |
| Other information | | |  |
| Funding | 22 | Give the source of funding and the role of the funders for the present study and, if applicable, for the original study on which the present article is based | 14 |

The International Statistical Classification of Disease 10 Code included in the analysis of Non-communicable Comorbidities in Pulmonary Tuberculosis and Healthcare Utilization: A Cross-Sectional Study of 2021 Indonesian National Health Insurance Data

| **Disease** | **ICD-10 Code** | **ICD10 Text** |
| --- | --- | --- |
| Respiratory tuberculosis | A15 | A15 Respiratory tuberculosis, bacteriologically and histologically confirmed |
| Cancer | C11 | C11 Malignant neoplasm of nasopharynx |
|  | C34 | C34 Malignant neoplasm of bronchus and lung |
|  | C38 | C38 Malignant neoplasm of heart, mediastinum, and pleura |
|  | C50 | C50 Malignant neoplasm of breast |
|  | C56 | C56 Malignant neoplasm of ovary |
|  | C73 | C73 Malignant neoplasm of the thyroid gland |
|  | C75 | C75 Malignant neoplasm of other endocrine glands and related structures |
|  | C76 | C76 Malignant neoplasm of other and ill-defined sites |
|  | C78 | C78 Secondary malignant neoplasm of respiratory and digestive organs |
|  | C79 | C79 Secondary malignant neoplasm of other and unspecified sites |
|  | C81 | C81 Hodgkin lymphoma |
|  | C85 | C85 Other and unspecified types of non-Hodgkin lymphoma |
|  | C91 | C91 Lymphoid leukemia |
| Diabetes Mellitus | E10 | E10 Type 1 diabetes mellitus |
|  | E11 | E11 Type 2 diabetes mellitus |
|  | E13 | E13 Other specified diabetes mellitus |
|  | E14 | E14 Unspecified diabetes mellitus |
| Mental Health Disease | F05 | F05 Delirium, not induced by alcohol and other psychoactive substances |
|  | F06 | F06 Other mental disorders due to brain damage and dysfunction and physical disease |
|  | F10 | F10 Mental and behavioral disorders due to the use of alcohol |
|  | F31 | F31 Bipolar affective disorder |
|  | F32 | F32 Depressive episode |
|  | F41 | F41 Other anxiety disorders |
|  | F45 | F45 Somatoform disorders |
|  | F50 | F50 Eating disorders |
|  | F79 | F79 Unspecified mental retardation |
|  | F80 | F80 Specific developmental disorders of speech and language |
|  | F88 | F88 Other disorders of psychological development |
|  | F89 | F89 Unspecified disorder of psychological development |
| Cardiovascular disease (CVD) | I05 | I05 Rheumatic mitral valve diseases |
|  | I07 | I07 Rheumatic tricuspid valve diseases |
|  | I09 | I09 Other rheumatic heart diseases |
|  | I10 | I10 Essential (primary) hypertension |
|  | I11 | I11 Hypertensive heart disease |
|  | I12 | I12 Hypertensive renal disease |
|  | I15 | I15 Secondary hypertension |
|  | I20 | I20 Angina pectoris |
|  | I21 | I21 Acute myocardial infarction |
|  | I24 | I24 Other acute ischaemic heart diseases |
|  | I25 | I25 Chronic ischaemic heart disease |
|  | I27 | I27 Other pulmonary heart diseases |
|  | I34 | I34 Nonrheumatic mitral valve disorders |
|  | I44 | I44 Atrioventricular and left bundle-branch block |
|  | I46 | I46 Cardiac arrest |
|  | I48 | I48 Atrial fibrillation and flutter |
|  | I49 | I49 Other cardiac arrhythmias |
|  | I50 | I50 Heart failure |
|  | I63 | I63 Cerebral infarction |
|  | I64 | I64 Stroke, not specified as hemorrhage or infarction |
|  | I69 | I69 Sequelae of cerebrovascular disease |
|  | I70 | I70 Atherosclerosis |
|  | I73 | I73 Other peripheral vascular diseases |
|  | I82 | I82 Other venous embolism and thrombosis |
|  | I87 | I87 Other disorders of veins |
|  | I88 | I88 Nonspecific lymphadenitis |
|  | I95 | I95 Hypotension |
| Chronic Obstructive Pulmonary Disease (COPD) | J40 | J40 Bronchitis, not specified as acute or chronic |
|  | J42 | J42 Unspecified chronic bronchitis |
|  | J43 | J43 Emphysema |
|  | J44 | J44 Other chronic obstructive pulmonary disease |
|  | J45 | J45 Asthma |
|  | J46 | J46 Status asthmaticus |
|  | J47 | J47 Bronchiectasis |
| Chronic Kidney Disease (CKD) | N04 | N04 Nephrotic syndrome |
|  | N08 | N08 Glomerular diseases |
|  | N11 | N11 Chronic tubulointerstitial nephritis |
|  | N13 | N13 Obstructive and reflux uropathy |
|  | N17 | N17 Acute renal failure |
|  | N18 | N18 Chronic kidney disease |
|  | N19 | N19 Unspecified kidney failure |
|  | N20 | N20 Calculus of kidney and ureter |
|  | N21 | N21 Calculus of Lower Urinary Tract |
|  | N23 | N23 Unspecified renal colic |
|  | N30 | N30 Cystitis |
|  | N36 | N36 Other disorders of the urethra |
|  | N39 | N39 Other disorders of the urinary system |
|  | N40 | N40 Hyperplasia of prostate |
|  | N49 | N49 Inflammatory disorders of male genital organs, not elsewhere classified |


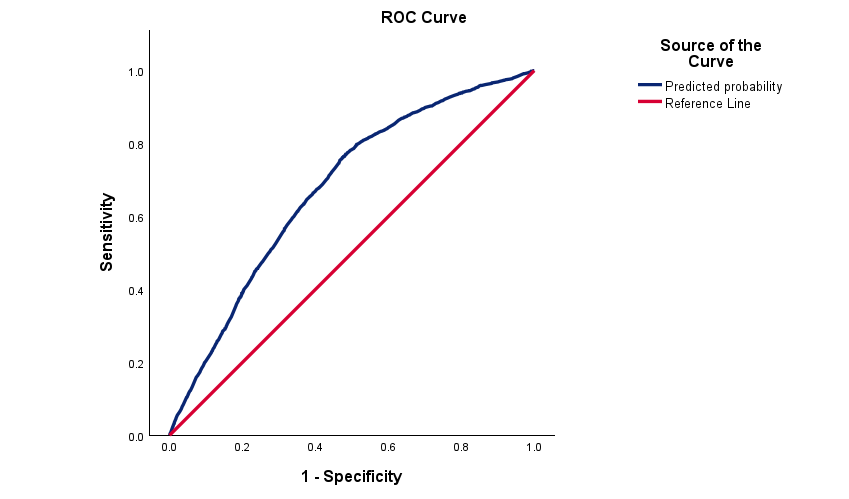


Receiver Operating Characteristic Result Associated Factor of Non-Communicable Disease Comorbidity among Indonesian National Health Insurance Users with Pulmonary Tuberculosis in 2021

|  | | | | |
| --- | --- | --- | --- | --- |
| Area Under Curve Associated Factor of Non-Communicable Disease Comorbidity among Indonesian National Health Insurance Users with Pulmonary Tuberculosis in 2021 | | | | |
| Area | Std. Error | Asymptotic Sig. ^b^ | Asymptotic 95% Confidence Interval | |
|  |  |  | Lower Bound | Upper Bound |
| .676 | .005 | .000 | .666 | .685 |


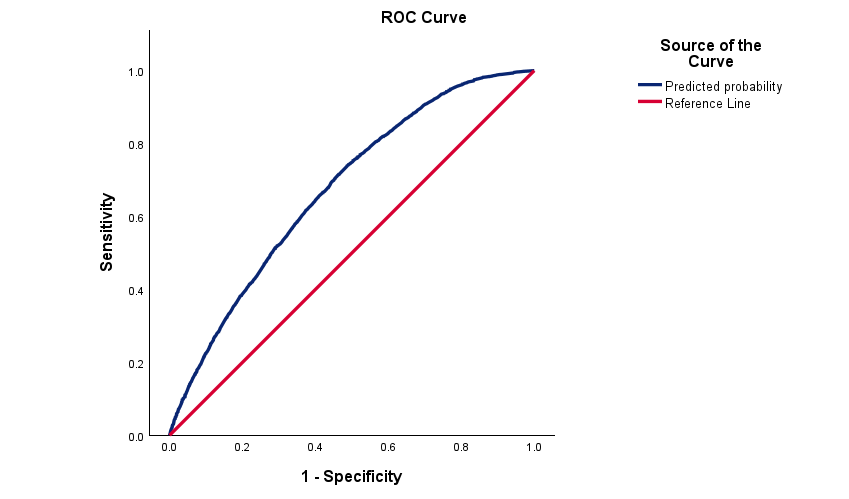


Receiver Operating Characteristic Result Associated Factor of Health Service Utilization among Indonesian National Health Insurance with Pulmonary Tuberculosis in 2021

| Area Under Curve Associated Factor of Health Service Utilization among Indonesian National Health Insurance Users with Pulmonary Tuberculosis in 2021 | | | | |
| --- | --- | --- | --- | --- |
| Area | Std. Error | Asymptotic Sig. ^b^ | Asymptotic 95% Confidence Interval | |
|  |  |  | Lower Bound | Upper Bound |
| .674 | .004 | .000 | .666 | .681 |
